# Orth GMN, Serpeloni F, Assis SG, Andrade TA, Rabe EM, Moura AAA. Mental disorders in adults deprived of liberty in American countries: a scoping review. Cad Saúde Pública 2025; 41(9):e00200624.

**DOI:** 10.1590/0102-311XER200624

**Published:** 2025-10-24

Where it reads:

Glaucia Mayara Niedermeyer Orth ^1^

Fernanda Serpeloni ^2^

Simone Gonçalves de Assis ^2^

Thais Afonso Andrade ^3^

Elisa Maria Rabe ^4^

Annelise Aurea Araújo de Moura ^5^

It should read:

Glaucia Mayara Niedermeyer Orth ^1^

Fernanda Serpeloni ^2^

Thais Afonso Andrade ^3^

Elisa Maria Rabe ^4^

Annelise Aurea Araújo de Moura ^5^

Simone Gonçalves de Assis ^2^

